# The potential of three whole blood microRNAs to predict outcome and monitor treatment response in sarcoid-bearing equids

**DOI:** 10.1007/s11259-022-09930-7

**Published:** 2022-04-28

**Authors:** E. Hamza, J. Cosandey, V. Gerber, C. Koch, L. Unger

**Affiliations:** 1 Swiss Institute of Equine Medicine, Department of Clinical Veterinary Medicine, Vetsuisse Faculty, University of Bern, Bern, Switzerland; 2 Departement of Zoonoses, Faculty of Veterinary Medicine, Cairo University, Cairo, Egypt

**Keywords:** Non-coding RNA, Biomarker, Equine oncology, Precision medicine

## Abstract

MicroRNAs (miRNAs) have been proposed as biomarkers for equine sarcoid (ES) disease. In this study, the suitability of three whole blood miRNAs to diagnose ES and to predict and monitor the outcome of therapy was explored. Using reverse transcription-quantitative polymerase chain reaction (RT-qPCR), expression levels of eca-miR-127, eca-miR-379, and eca-miR-432 in whole blood of ES-affected equids before and at least one year after therapy were compared to those of unaffected control equids. Associations of age, sex, species, diagnosis, and therapy outcome with miRNA expression levels were examined using general linear models. In total, 48 ES-affected equids and 47 control equids were recruited. From the affected animals, 31 responded favorably to treatment, and 17 demonstrated a failure of therapy. None of the tested miRNAs were influenced by age. Male equids showed increased expression of eca-miR-127 compared to females and horses showed higher expression levels of eca-miR-379 and eca-miR-432 than donkeys. Eca-miR-127 was confirmed as a diagnostic discriminator between ES-affected and control equids. No difference in miRNA profiles before therapy was found when comparing ES-affected equids with success vs. failure of therapy. Eca-miR-379 and eca-miR-432 decreased over time in horses where therapy was successful, but not in those cases where it failed. Biological variables influence equine whole blood miRNA expression, which may complicate biomarker validation. While none of the tested miRNAs could predict the response to therapy in ES-affected equids and eca-miR-127 showed poor diagnostic accuracy for ES, eca-miR-379 and eca-miR-432 miRNAs might allow refinement of monitoring of success of ES therapy.

**Supplementary Information:**

The online version contains supplementary material available at 10.1007/s11259-022-09930-7.

## Introduction

Equine sarcoids are the most common equine skin tumors in horses and donkeys (Valentine 2006; Schaffer et al. 2013). Besides the main etiological agents, bovine papillomavirus 1, 2 and potentially 13 (BPV 1, 2 and 13), a genetic predisposition with a proposed polygenic basis should be considered as well as external factors like trauma (Chambers et al. 2003; Knottenbelt 2006; Lunardi et al. 2013; Christen et al. 2014; Staiger et al. 2016). Additionally miRNAs seem to play a role in the pathogenesis of this multifactorial disease (Pawlina et al. 2017; Terron-Canedo et al. 2017).

MiRNAs are small, 20 to 24 nucleotide long, non-coding RNA molecules that target messenger RNAs (mRNAs), induce mRNA degradation and translational repression, and thus ultimately lead to downregulation of gene expression (O’Brien et al. 2018). For various species including horses, it has been shown that miRNAs are expressed on the tissue level in a tissue-dependent manner (cellular miRNAs) and can also be quantified in the circulation (circulating cell-free miRNAs) (Ludwig et al. 2016; Pacholewska et al. 2016; Unger et al. 2019a; Rahat et al. 2020). Circulating miRNAs are proposed as potential epigenetic markers for precision medicine in humans. Particularly in the field of oncology, the potential of so-called miRNA-based liquid biopsies is being currently explored, aiming to establish an early diagnosis, determine cancer subtypes, and predict the overall outcome and response to therapy (Rahat et al. 2020; Larson et al. 2021).

We have previously proposed selected serum and whole blood miRNAs to provide a non-invasive method to diagnose ES (Unger et al. 2019a, 2019b, 2021; Cosandey et al. 2021). However, during the validation phase, we identified multiple factors that can bias equine circulating miRNA expression, most importantly biological variables such as breed, sex, and age, and in the case of serum samples additional pre-analytical factors such as hemolysis (Unger et al. 2019a, 2021; Cosandey et al. 2021). In whole blood, for example, male equids had higher miRNA levels compared to female equids and some miRNA biomarker candidates were breed- or sex-specific, which limits their use as universally applicable diagnostic tools for ES in equine practice (Cosandey et al. 2021).

The goal of this study was to assess the diagnostic and prognostic properties of three whole blood miRNAs that have recently been suggested as biomarkers for ES disease in an additional independent study cohort (Unger et al. 2019b; Cosandey et al. 2021). Eca-miR-127, eca-miR-379, and eca-miR-432, are all encoded by a large miRNA cluster on equine chromosome 24, which corresponds to the well-documented, predominantly tumor-suppressive human 14q32 miRNA cluster, and has previously been proposed to play a role in ES pathogenesis (Zehavi et al. 2012; Bogedale et al. 2019; Unger et al. 2019b). Specifically, we aimed to evaluate the suitability of the selected miRNAs to assist in the diagnosis of ES, predict the outcome and monitor ES therapy. Importantly, to detect potential bias limiting their clinical usefulness, the influence of biological variables on the miRNA expression levels was assessed in parallel.

## Material and methods

### Study design and study cohort

For this retrospective case–control study, we reviewed the medical records of cases presented between January 2015 and December 2019 to the Swiss Institute of Equine Medicine (ISME) equine clinic for treatment of ES, of which a whole blood sample taken prior to therapy was stored at -80 °C in the ISME bio archive. Inclusion criteria were 1) a histologically confirmed diagnosis of ES disease, 2) a complete clinical description of ES lesions and 3) either a clinical follow-up exam at least one year after the first examination or euthanasia due to deterioration of ES disease by a veterinarian in the field. A second whole blood sample was collected at the time of the follow-up examination. Cases were excluded if the histological diagnosis was equivocal, if no initial blood sample was available, if other types of tumors were present, or non-presentation of the equid for follow-up for reasons other than euthanasia due to deterioration of the ES lesions. The complete clinical description at the first and, if available, the follow-up examination included the localization, number, and types of ES lesions, as well as a disease severity score ranging from 3 (mild lesions) to 19 (severe lesions) (Mählmann et al. 2014).

The disease severity scores of the first and follow-up examination were used to define the response to treatment, which was broadly divided into success vs. failure. Equids which showed a complete remission (score at second examination = 0) or substantial improvement (score at follow-up examination decreased by at least 2 points compared to first examination, which corresponds to a substantial improvement in terms of total surface area, worst clinical type, number or growth rate of ES lesions) were categorized as a treatment success. Cases with no substantial improvement (score at the follow-up examination ± 1 compared to the first examination) or whose score at the follow-up examination increased by at least 2 points compared to the first examination, corresponding to obvious worsening of ES lesions, were included in the group of equids with failed therapy.

The control group consisted of privately owned equids that based on the diagnosis in the clinical records were presented for non-neoplastic conditions, equids that stayed in the hospital as companions for other cases, and teaching horses owned by ISME. Control equids had to be free of skin tumors and other dermatological problems and show no signs of internal neoplastic disorders. Controls were selected by individual matching to cases based on the variables: species (horse or donkey), breed (the same or a closely related breed), age (± 3 years), and sex (female or male including stallions and geldings). Controls were only included if at least 2 out of the 3 variables matched.

The owners of all animals included in the study gave their informed consent that blood samples obtained by venipuncture for diagnostic or therapeutic purposes could also be used for research. Additional samples from cases and controls were collected during experiments approved by the Animal Experimentation Committee of the Canton of Bern, Switzerland (BE110/15, BE41/19 +).

### Sample collection and storage

Blood was collected from the jugular vein into ethylenediamine tetraacetic acid (EDTA) tubes, then transferred to cryotubes (Sarstedt, Nümbrecht, Germany) and stored at − 80 °C in the ISME bio archive.

### Preanalytics

RNA was extracted from the whole blood samples using PAXgene Blood miRNA Kit (Qiagen, Hombrechtikon, Switzerland) according to a previously established protocol (Unger et al. 2016). RNA-concentrations and RNA Integrity Numbers (RIN) were measured with a Fragment Analyzer and the Standard Sensitivity RNA Analysis Kit (Advanced Analytical, Heidelberg, Germany). The extracted RNA was stored at -80 °C.

### RT-qPCR

Eca-miR-127, eca-miR-379, and eca-miR-432 were chosen as candidate miRNAs based on their potential prognostic and/ or diagnostic properties shown in two previous studies (Unger et al. 2019b; Cosandey et al. 2021). Eca-miR-30d was selected as an endogenous control and cel-miR-39-3p as an exogenous control as previously recommended (Cosandey et al. 2021). Information on miRNA primers and probes have been published elsewhere (Cosandey et al. 2021). The RT- and qPCR reactions were performed as previously described. Briefly, the RT-reactions were carried out using the TaqMan MicroRNA Reverse Transcription Kit (ThermoFisher, Reinach, Switzerland) and sequence-specific stem-loop RT-primers for the candidate miRNAs and endogenous and exogenous controls (ThermoFisher, Reinach, Switzerland). The qPCR reactions containing specific primers and the RT product were run in triplicates in a 7300 Real-Time PCR system (Applied Biosystems, CA, USA) (Cosandey et al. 2021). For assay quality assessment, the raw cycle quantification (Cq) values of the tested miRNAs were compared between the three technical replicates for each candidate miRNA and in each sample. A maximum Cq difference of 0.5 was accepted. If the difference was > 0.5, the Cq of the corresponding technical replicate was excluded. For data normalization, the 2^−ΔΔCq^ method, a previously published relative quantification method, was applied (Cosandey et al. 2021).

### Statistics

Statistical analysis was performed using NCSS 12 software (NCSS, Kaysville, Utah).

Descriptive statistics are given as mean ± SD and ranges. To assess if matching was appropriate, the distribution of age groups (young = 0–8 years, middle-aged = 9–20 years, old > 20 years) (Lehnhard et al. 2004) and sex (males = geldings and stallions vs. females = mares) was compared in ES-affected vs. ES-free horses and donkeys using Fisher’s exact test. The breed distribution in the study cohort was not evaluated since it was deemed too diverse to be combined into meaningful subgroups. Wilcoxon signed-rank test was used to compare disease severity scores of ES-affected equids at the time of the first and second examination.

The normality tests (Shapiro–Wilk W, Kolmogorov–Smirnov, D’Agostino Skewness, D’Agostino Kurtosis, D’Agostino Omnibus) showed that normalized miRNA expression data were not normally distributed. Therefore, the data were log-transformed, and Grubb’s test was used to detect outliers for each candidate miRNA in ES-affected and ES-free equids separately. If outlier data points from at least one candidate miRNA were identified for a sample of a study subject, this sample was excluded from further analysis.

A General Linear Model (GLM) was used to examine the influence of species (horses vs. donkeys), age groups, sex, and diagnosis (ES-affected vs. ES-free equids) and their interactions (diagnosis and species, diagnosis and sex) on the expression of the candidate miRNAs at the time of the first examination. The interaction of age with other factors was not examined due to the small number of individuals per age group when combined with other factors. As mentioned above, the breed distribution was too diverse in the study cohort to allow grouping in breed categories. Thus, the influence of the biological variable breed on miRNA expression was not further assessed. The influence of ES type or number on miRNA expression was not further evaluated since the majority of ES bearing equids suffered from more than one single ES type and had multiple ES lesions.

In a second GLM, the potential of the three candidate miRNAs to predict the outcome of therapy (failure vs. success) in whole blood samples of ES affected equids taken at the time of the first examination was evaluated. The effects of sex and species and their interactions with the outcome were assessed as described above. For each of the three candidate miRNAs, p-values ≤ 0.05 were adjusted for multiple testing by the Benjamini–Hochberg method, and only considered significant if they were smaller than the adjusted p-value.

When a factor reached significance in the GLMs, the expression of the corresponding miRNA was compared between the respective groups using Mann–Whitney U test as the post-hoc test. For miRNAs with significant differences in expression between groups, an area under the curve (AUC) receiver operating characteristic (ROC) curve analysis was performed allowing the calculation of their specificity and sensitivity as diagnostic and/ or prognostic biomarkers for ES disease. Additionally, the positive likelihood ratio (LR +), negative likelihood ratio (LR-), diagnostic odds ratio (DOR), pre- (PRP), and post-test probability (POP) were calculated (Eusebi 2013).

Wilcoxon signed-rank tests were performed to compare miRNA expression at the time of the first vs. follow-up examination overall and after subgrouping depending on the outcome of therapy (success vs. failure).

In all analyses, *p*-values ≤ 0.05 were considered significant.

All figures were created using NCSS 12 software (NCSS, Kaysville, Utah).

## Results

### Study cohort

In total, 87 equids with ES disease and 102 ES-free control equids from the ISME bio archive were assessed for eligibility. Of those, 48 ES-affected equids consisting of 37 horses and 11 donkeys with a mean ± SD (range) age of 9.7 ± 5.1 (3–24) years met the inclusion criteria. Reasons for exclusion from the study are shown in Supplementary Fig. 1. Detailed information on breed, sex, and age of the individual ES-affected equids, on localization, type and number of ES lesions, and disease severity scores at the time of the first and follow-up examination as well as on type and outcome of therapy is given in Supplementary Table 1a. Summary information of the distribution of sex, age groups, type, and outcome of therapy in the entire group of ES-affected equids are depicted in Table 1a. 13 equids (9 horses and 4 donkeys) had at least one further ES treatment (mean 1.2 ± 0.6, range: 1–3) before the follow-up examination (Supplementary Table 1a, Supplementary Table 2). Four horses (1831, 2585, 2782) and one donkey (1009) had ES treatments in the ISME equine clinic before inclusion in the study and one horse (2060) was treated for ES after the follow-up examination (Status December 2021) (Supplementary Table 2). Information on the types of additional ES treatments is given in Supplementary Tables 1a and 2. Three donkeys (1009, 2566, 2061) were euthanized due to deterioration of ES disease before a standardized follow-up examination could be performed. For 65% (31/48) of all ES-affected equids therapy was successful, whereas for 35% (17/48), a failure of therapy was reported. The time between the first and the second examination was 3.2 ± 1.1 (1.1–5.0) years. The disease severity scores of ES-affected equids with a follow-up examination decreased significantly from the first to the follow-up examination (*p* < 0.001), with scores of 13.2 ± 3.4 (5–18) at the first examination compared to 6.6 ± 7.4 (0–19) at the follow-up examination. In one horse (2262) and one donkey (2352), a second blood sample could not be collected at the follow-up examination due to uncooperative behavior reducing the number of follow-up whole blood samples to 43.

**Table 1:** Descriptive statistics study cohort. Age categories: 0–8 years = young, 9–20 years = middle, > 20 = old. Types of therapies throughout the study period: excision = only surgical excision, chemotherapy = only chemotherapy, combined therapy = surgical excision in combination with chemotherapy and/ or immunotherapy. Outcome: success = complete (disease severity score/ DSS at follow-up examination = 0) or partial remission (DSS at follow-up examination decreased by ≥ 2 points compared to DSS at 1^st^ examination), failure = no substantial improvement (DSS at follow-up examination =  ± 1 points compared to 1^st^ examination) or clear deterioration (DSS at follow-up examination increased by ≥ 2 points compared to DSS at 1^st^ examination

Variable	Group	Horses	Donkeys	Total
**A**: Distribution of sex, age, type and outcome of therapy in the group of ES-affected equids				
Sex	Male	25/37 (67.6%)	6/11 (54.5%)	31/48 (64.6%)
	Female	12/37 (32.4%)	5/11 (45.5%)	17/48 (35.4%)
Age	Young	15/37 (40.5%)	6/11 (54.5%)	21/48 (43.8%)
	Middle	19/37 (51.4%)	5/11 (45.5%)	24/48 (50%)
	Old	3/37 (8.1%)	0/11 (0%)	3/48 (6.3%)
Type of therapy	Excision	23/37 (62.2%)	10/11 (90.9%)	33/48 (68.8%)
	Chemotherapy	1/37 (2.7%)	0/11 (0%)	1/48 (2.0%)
	Combined	13/37 (35.1%)	1/11 (9.1%)	14/48 (29.2%)
Outcome	Success	24/37 (64.9%)	7/11 (63.6%)	31/48 (64.6%)
	Failure	13/37 (35.1%)	4/11 (36.4%)	17/48 (35.4%)
**B:** Distribution of sex and age in the group of ES-free control equids				
Sex	Male	25/37 (67.6%)	6/10 (60%)	31/47 (66%)
	Female	12/37 (32.4%)	4/10 (40%)	16/47 (34%)
Age	Young	17/37 (45.9%)	2/10 (20%)	19/47 (40.4%)
	Middle	18/37 (48.6%)	8/10 (80%)	26/47 (55.3%)
	Old	2/37 (5.4%)	0/10 (0%)	2/47 (4.3%)

For individual matching based on the criteria species, age, sex, and breed as defined above, 47 out of 102 ES-free control equids consisting of 37 horses and 10 donkeys with an age of 10.6 ± 4.8 (2–24) years were selected. Detailed information on breed, sex, age, and diagnoses of the individual control equids is given in Supplementary Table 1b and summary information on age and sex distribution in the entire group of control equids in Table 1b. For one donkey (2566), no matching control could be identified in our dataset. For five equids matching with their corresponding controls was not possible for all three criteria with imperfect matching for age (2927, 2945, 2337, 2557) and sex (2558). However, age and sex distributions did not significantly differ between ES-affected vs. ES-free equids, ES-affected vs. ES-free horses, and ES-affected vs. ES-free donkeys.

### Preanalytics

In all whole blood samples, the RNA concentration was 21.5 ± 20.3 (0.2–91) ng/μL. RINs were 4.3 ± 2.7 (1–10) (Supplementary Table 3). Based on the results of previous studies, we considered the RNA quantity and quality of all samples to be sufficient for RT-qPCR (Unger et al. 2016, 2019b).

### Reverse transcription quantitative PCR

The raw Cq values and the 2^−ΔΔCq^ values of the three tested candidate miRNAs are depicted in Supplementary Table 4. Three samples from the follow-up examination (2906, 2879, 2938) were excluded from further analysis because they were showing one or more outlier data points using Grubb’s test (Supplementary Table 4).

To assess the diagnostic and prognostic potential of the three miRNAs examined in whole blood samples taken at the first presentation, the significance levels (p-values) of diagnosis and outcome, respectively, and their interactions with biological variables were tested using the GLMs (Table 2a and b).

**Table 2:** Diagnostic and prognostic potential of eca-miR-127, eca-miR-379, and eca-miR-432 and interaction with biological variables. Statistically significant p-values (≤ 0.05) are labeled with an asterisk* and the adjusted p-values (p adj) are indicated

**A:** MiRNA expression at the first presentation – p-values of the effects of diagnosis, species, and sex and their interactions					
eca-miR	Diagnosis	Species	Interaction diagnosis and species	Sex	Interaction diagnosis and sex
-127	0.02* p adj 0.05	0.30	0.63	0.005* p adj 0.05	0.98
-379	0.55	< 0.001* p adj 0.03	0.67	0.08	0.63
-432	0.72	< 0.001* p adj 0.03	0.79	0.82	0.70
**B:** MiRNA expression at the first presentation – p-values of the effects of outcome, species and sex and their interactions					
eca-miR	Outcome	Species	Interaction outcome and species	Sex	Interaction outcome and sex
-127	0.28	0.30	0.84	0.007* p adj 0.05	0.98
-379	0.47	0.002* p adj 0.05	0.72	0.16	0.99
-432	0.72	< 0.001* p adj 0.03	0.73	0.98	0.71

**Fig. 1:**
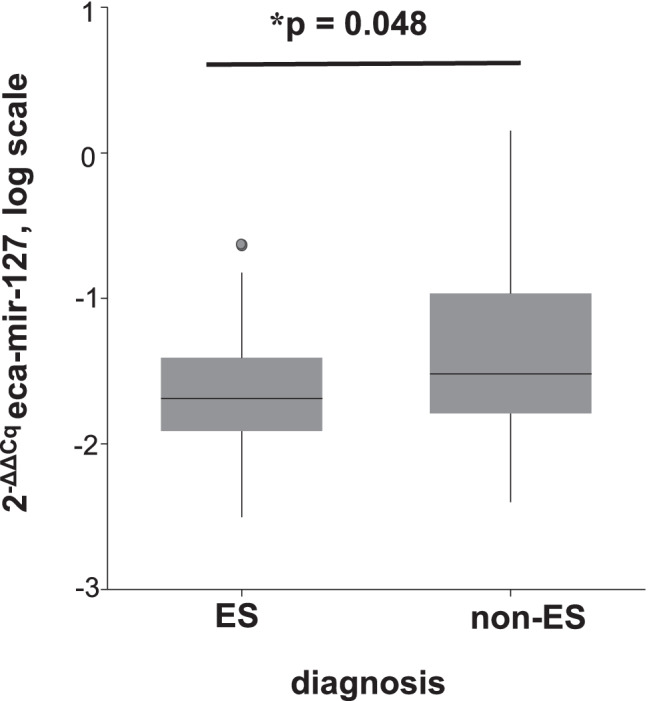
Box plot depicting the influence of diagnosis (ES vs. ES-free) on whole blood expression of eca-miR-127 in all equids at the time of the first examination. The y-axis shows the 2-ΔΔCq values of the candidate miRNA on a log scale. Significant p-values are marked with an asterisk (*)

**Fig. 2:**
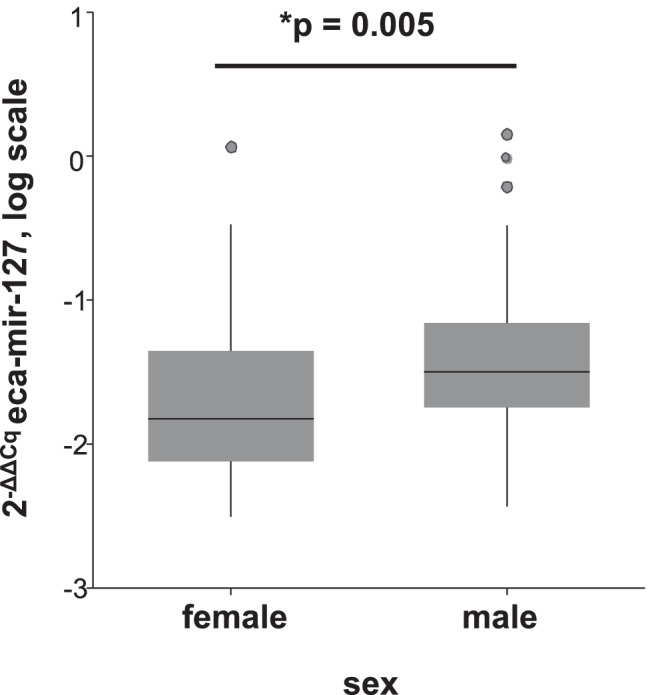
Box plot depicting the influence of sex (male vs. female) on whole blood expression of eca-miR-127 in all equids at the time of the first examination. The y-axis shows the 2-ΔΔCq values of the candidate miRNA on a log scale. Significant p-values are marked with an asterisk (*)

**Fig. 3:**
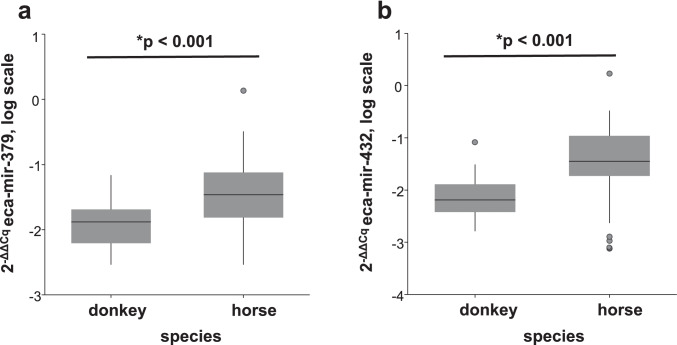
Box plots depicting the influence of species (horse vs. donkey) on whole blood expression of eca-miR-379 (**a**) and eca-miR-432 (**b**) in all equids at the time of the first examination. The y-axis shows the 2-ΔΔCq values of the candidate miRNAs on a log scale. Significant p-values are marked with an asterisk (*)

**Fig. 4:**
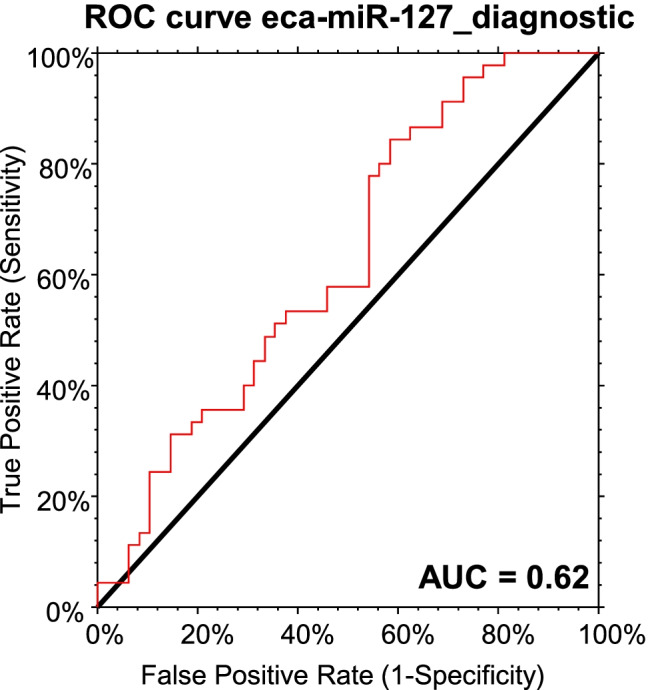
ROC curve analysis assessing whole blood expression of eca-miR-127 for discrimination of ES- affected from ES-free equids at the time of the first examination. Abbreviations: ROC = Receiver Operating Characteristic, AUC = area under the curve

In the first GLM, out of the three tested miRNAs, only eca-miR-127 was significantly associated with diagnosis of ES disease (GLM: *p* = 0.02). ES-affected equids had lower expression of eca-miR-127 than ES-free equids (Mann–Whitney U test: *p* = 0.048) (Fig. 1). Sex had a significant effect on eca-miR-127 expression levels (GLM: *p* = 0.005), but the interaction of diagnosis and sex was not significant (Table 2a). The expression levels of eca-miR-379 and eca-miR-432 were not influenced by sex but by the factor species (GLM: *p* < 0.001 and *p* < 0.001, respectively) (Table 2a). Male equids showed higher levels of eca-miR-127 in whole blood than female equids (Mann–Whitney U test p = 0.005) and horses showed higher expression of eca-miR-379 and eca-miR-432 compared to donkeys (Mann–Whitney U test *p* < 0.001and *p* < 0.001, respectively) (Fig. 2, Fig. 3 and b). The ROC curve analysis indicated an AUC of 0.62 (95% CI = 0.49–0.72, *p* = 0.04), with a sensitivity of 57% (95% CI = 42–72%) and a specificity of 54% (95% CI = 39–68%) for whole blood eca-miR-127 to diagnose ES in the whole study population (Fig. 4). The LR + and LR − for eca-miR-127 to diagnose ES were 1.3 and 0.8, respectively, with a DOR of 1.6, a PRP of 49%, and a POP of 55%.

In the second GLM, none of the tested miRNAs were significantly associated with the outcome of ES disease (Table 2b). Also, when evaluating the prognostic potential of the three tested miRNAs, a significant influence of biological variables on miRNA expression was found: Eca-miR-127 was again influenced by the factor sex (GLM: *p* = 0.007) and eca-miR-379 and eca-miR-432 were influenced by the factor species (GLM: *p* = 0.002 and *p* < 0.001, respectively) with similar expression trends as found in the first GLM (Table 2b).

**Fig. 5:**
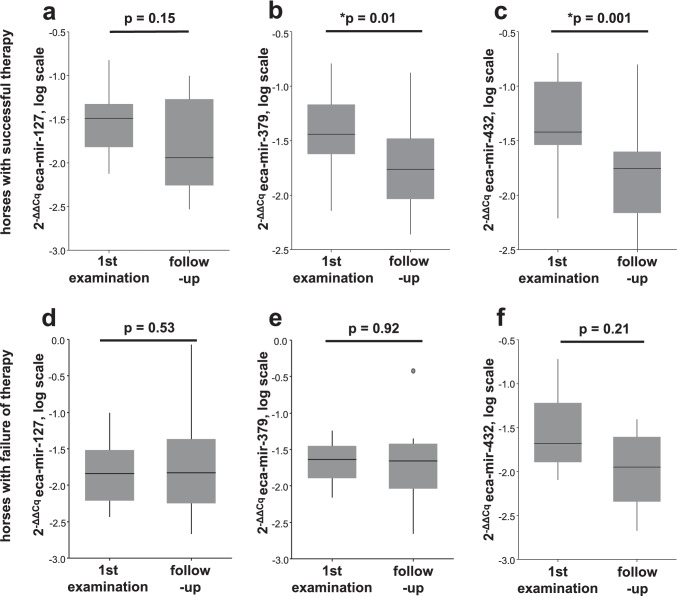
Box plots depicting the comparison of expression of whole blood eca-miR-127 (A, D), eca-miR-379 (**b, e**) and eca-mir-432 (**c, f**) at the time of the first and the second examination in horses with successful therapy (**a-c**) and in horses with failure of therapy (**d-f**). The y-axis shows the 2-ΔΔCq values of the candidate miRNAs on a log scale. Significant p-values are marked with an asterisk (*)

Whole blood miRNA expression levels at the time of the first and a follow-up examination were only compared in horses, since only seven donkeys had a second blood sample available for miRNA analysis. Of those, we excluded one sample (2906) with outlier data points from further analysis. From the remaining six donkeys, five showed success and one a failure of therapy, which made comparisons based on the outcome of therapy impossible in this species. In horses, eca-miR-379 and eca-miR-432 showed overall lower expression levels at the time of the follow-up examination (Wilcoxon signed-rank test: *p* = 0.03 and *p* < 0.001, respectively) (Table 3). In the group of horses with successful therapy, these differences remained significant (Wilcoxon signed-rank test, eca-miR-379: *p* = 0.01 and eca-miR-432: *p* = 0.001) (Table 3, Fig. 5a-c), whereas in the group of horses with failure of therapy no statistically significant differences were found (Table 3, Fig. 5d-f).

**Table 3:** Differences in miRNA expression compared between first and follow-up examination in the group of ES-affected horses. Statistically significant p-values (≤ 0.05) are labeled with an asterisk*

eca-miR	ES-affected horses	ES-affected horses with successful therapy	ES-affected horses with failure of therapy
-127	0.14	0.15	0.53
-379	0.03*	0.01*	0.93
-432	< 0.001*	0.001*	0.21

## Discussion

This study provides further evidence that equine whole blood miRNAs are potential diagnostic biomarkers for ES disease. Furthermore, we showed for the first time that quantification of miRNAs may help to monitor the success of ES therapy. However, none of the tested miRNAs were able to predict response to therapy based on the expression levels before initiation of treatment. The identification of universally applicable miRNA biomarkers was complicated by the influence of biological variables on whole blood miRNA fingerprints.

Similar to the findings of a previous study, the expression of eca-miR-127 was influenced by sex, although sex had no influence on the use of this miRNA as a diagnostic biomarker (Cosandey et al. 2021). In contrast to previous results, sex did not impact the expression of eca-miR-379 and eca-miR-432 (Cosandey et al. 2021). Sex-biased miRNA signatures have been described in different avian and mammalian species, including horses (Meder et al. 2014; Selvamani et al. 2014; Warnefors et al. 2017; Cui et al. 2018; Fehlmann et al. 2020; Cosandey et al. 2021; Piscopo et al. 2021). In a large-scale miRNA analysis using various human tissues including peripheral blood, 14% of the tested miRNAs turned out to be sex-biased and to be significantly more tissue-specific compared to non-biased miRNAs (Cui et al. 2018). Some miRNAs may even show opposite expression patterns between females and males (Guo et al. 2017). Ignoring sex-related differences in miRNA expression may introduce bias in biomarker studies (Piscopo et al. 2021). Breed-specific equine miRNA expression has been previously described, and shown to have an impact on the use of eca-miR-127 as a diagnostic biomarker for ES disease (Barrey et al. 2010; Pacholewska et al. 2016; Cosandey et al. 2021). Because of the highly heterogeneous breed distribution, breed could not be assessed as a possible confounding factor, which is a limitation of our study. Nonetheless, special care was taken to ensure adequate matching of ES cases and controls to reach similar biological variation in both groups of equids.

Research on miRNA expression in donkeys is still in its infancy. Only a few studies have investigated asinine miRNA fingerprints in testis, cauda epididymis, and milk (Tian et al. 2020; Mecocci et al. 2021). This is the first study to show differences in miRNA fingerprints between horses and donkeys. In a previous study, horses and donkeys showed similar expression of miRNAs in serum (Unger et al. 2021). However, both studies investigated only selected miRNAs in the circulation. A larger-scale next-generation sequencing experiment could help to unravel general differences and similarities of miRNA expression in the two related species.

Histopathology of tumor tissue specimens remains the gold standard for ES diagnosis. This however requires a biopsy to be taken, which in turn may lead to disease aggravation and is thus often avoided in a clinical setting (Knottenbelt and Kelly 2000). Furthermore, histological differentiation of ES from other types of spindle cell tumors can be challenging, even when combined with BPV-1 and -2 PCR testing (Martens et al. 2000; Epperson and Castleman 2017). Recently, fibroblast-associated protein-α (FAPα) labeling was confirmed in ES lesions and suggested as a novel marker for immunohistological diagnosis of ES (Tura et al. 2021). Due to the aforementioned limitations of histopathological diagnosis, ES are most commonly diagnosed clinically with a specificity and sensitivity of around 80%. However, depending on the level of clinical experience, up to 30% of ES-like skin lesions may be misclassified as ES which may ultimately lead to the wrong choice of therapy (Koch et al. 2018). Collection of swabs or scrapings from the surface of lesions suspicious for ES for subsequent BPV-1 and -2 PCR testing can aid in the non-invasive diagnosis of ES disease but may lack sensitivity if minimal epidermal changes are present or BPV loads are low (Martens et al. 2001; Bogaert et al. 2008). Serum and whole blood miRNAs have been recently proposed as potential adjunct non-invasive diagnostic tools for challenging ES cases (Unger et al. 2019a).

In a previous study, serum eca-miR-331 had a sensitivity of 60% and a specificity of 71% to diagnose ES and its expression was neither influenced by biological variation and ES type or disease severity nor by hemolysis. However, it was not able to discriminate between horses with ES and other types of skin tumors (Unger et al. 2021). Whole blood eca-miR-127 was only a weak diagnostic discriminator between ES-affected and ES-free equids in this and a previous study with sensitivities and specificities around or even below 60% (Cosandey et al. 2021). In this study, the probability of an equid having ES disease increased only from 49 to 55% with a positive test result. Interestingly, the diagnostic power of eca-miR-127 appears to be highly dependent on the biological variable "breed": In Franches-Montagnes horses this miRNA biomarker reached a sensitivity of 91% and a specificity of 83% to diagnose ES, whereas in Swiss Warmblood horses it turned out to be non-diagnostic (Cosandey et al. 2021). However, for the reasons discussed above, the variable "breed" could not be included in the analyses (e.g. only four Franches-Montagnes horses in the case cohort, Supplementary Table 1a). Regardless, breed-specific biomarkers are of limited use in clinical settings, where universally applicable biomarkers are preferred.

In this study, the difference in disease severity scores between the first and the follow-up examination was used to classify the response to therapy as either success or failure. This appeared to us as the most logical and objective way to assess the success of therapy with the available data. However, in a clinical setting, the distinction of success and failure heavily depends on the concrete objective of the treatment approach in every individual case. Therefore, the chosen assessment regarding the response to therapy may be hampered, particularly in severely affected equids with multiple lesions covering large areas of the integument. In such cases, the more realistic aim of therapy is rarely a massive reduction of the affected surface area, but often just to eliminate large bleeding, ulcerated tumors or tumors in troublesome areas, such as the eyelids, where they may limit the lid function, or in the girth area or at the base of the ear, where they impede the use under saddle. However, the strength of our study lies in being able to closely follow ES cases for between one and five years and the clinical impression in terms of the success of the therapy largely reflected the classification used in this study. The choice of therapy is clearly influenced by factors such as localization or extent of ES tumors that limits or precludes the possibility of surgical removal or owner compliance in case of topical treatments that are often continued at home. In this study, we did not categorize equids depending on the type of therapy applied, because advanced cases in particular were often presented on numerous occasions and diverse treatment modalities were applied. In addition, we cannot exclude with certainty that additional treatments were performed elsewhere. The points mentioned above vividly illustrate the complexity of ES therapy and the assessment of therapeutic success.

In contrast to the various available invasive and non-invasive tools to aid with diagnosing ES-disease, there is currently no clinically applicable test to help prognosticate the clinical progression of ES disease and response to therapy. Selected miRNAs from our initial prognostic biomarker discovery tested in this and a previous validation study were not confirmed as predictors of disease outcome in ES-affected equids after therapeutic intervention (Unger et al. 2019b; Cosandey et al. 2021). In contrast, we recently identified two whole blood miRNAs, eca-miR-432 and eca-miR-125a-5p, that were able to predict if young male (but not female) ES-free horses will spontaneously develop ES lesions within the next 5–12 years with satisfactory sensitivities and specificities around 70 to 80%. For mares, no such prognostic miRNA biomarker could be identified (Cosandey et al. 2021). Sex-specific biomarkers may gain more importance in the future for characterizing subgroups of patients and tailoring the best therapeutic approach for the individual patient (Piscopo et al. 2021).

In the current study, whole blood expression of eca-miR-379 and eca-miR-432 decreased over time in horses with successful therapy, but not in those where it failed. These miRNAs may thus qualify as biomarkers to monitor response to ES therapy. The clinical value of such biomarkers is obviously limited in patients with purely external skin tumors, that do not metastasize to internal organs, since the course of the disease can be easily assessed from the outside. However, the results of this study support the investigation of miRNA blood biomarkers in other types of equine tumors such as lymphomas or melanomas, where clinical monitoring of disease progression is more limited. Dynamic changes in miRNA trends in response to therapy have been associated with the outcome of antitumor therapy in people (Ponomaryova et al. 2016). In cholangiocarcinoma cases, it has been shown that preoperative miRNA levels were not suitable for prediction of survival. However, a strong postoperative decline of hsa-miR-122 was associated with a more favorable prognosis (Loosen et al. 2019). Further studies including larger study cohorts are warranted to confirm a direct link of dynamic miRNA expression over time with the outcome of ES disease and other neoplastic disorders.

In the course of our equine miRNA biomarker research, the results of the initial next-generation sequencing-based discovery studies were not completely reproducible in the validation studies using larger and more diverse study cohorts and RT-qPCR for miRNA detection and quantification (Unger et al. 2019a, 2019b, 2021; Cosandey et al. 2021). This is a commonly observed phenomenon in cancer biomarker research. Only a few biomarkers can be translated from the initial discovery stage to clinical application (Goossens et al. 2015; Keller and Meese 2016). As for miRNA biomarkers, discrepancies between discovery and validation studies are mostly owed to the inclusion of a small number of study subjects in the discovery phase, different methods used for miRNA quantification and a general lack of standardized miRNA processing and data analysis including normalization strategies (Faraldi et al. 2018; Kok et al. 2018). Furthermore, the influence of biological variables such as age or sex on circulating miRNA expression is often ignored in the discovery phases (Piscopo et al. 2021). Despite extensive research for almost two decades, only a few and mainly cellular miRNA-based tests have been confirmed as diagnostic biomarkers for neoplastic conditions in a clinical settings (Brand et al. 2014; Labourier et al. 2015; Lithwick-Yanai et al. 2017; Bonneau et al. 2019). However, great effort is currently being made to conduct large-scale validation trials with the aim to market blood-based miRNA tests for early cancer diagnosis in humans (Hummingbird Diagnostics 2022; Toray 2022). Studies including similarly large numbers of subjects are unfortunately often difficult to implement in equine medicine. In the future, more focus should be put on equine multi-center miRNA research projects. Furthermore, combinations of different miRNA expression levels should be investigated since panels composed of several miRNAs may have greater diagnostic power than single miRNAs (Mariani et al. 2021).

## Conclusions

While none of the tested miRNAs were able to predict response to therapy in ES-affected equids, they still showed potential to inform personalized treatment decisions in the future. Eca-miR-127 was confirmed as a diagnostic predictor for ES in equids, albeit with poor sensitivity and specificity. Eca-miR-379 and -432 decreased in ES-affected horses that responded favorably to therapy and might be investigated in further studies as potential biomarkers for monitoring treatment interventions in ES disease.

## Supplementary Information

Below is the link to the electronic supplementary material.

Supplementary file1 (XLSX 28 KB)

Supplementary file2 (XLSX 13 KB)

Supplementary file3 (XLSX 13 KB)

Supplementary file4 (XLSX 118 KB)

Supplementary file5 (PDF 80.4 KB)

## Data Availability

All data generated or analyzed during this study are included in this published article and its supplementary information files.
