# Supplementary figures and images for: The potential of three whole blood microRNAs to predict outcome and monitor treatment response in sarcoid-bearing equids
Source: Vet Res Commun. 2022 Apr 28;47(1):87–98. doi: 10.1007/s11259-022-09930-7 (PMC9873782; doi:10.1007/s11259-022-09930-7)

Supplementary Figure 1: Case recruitment

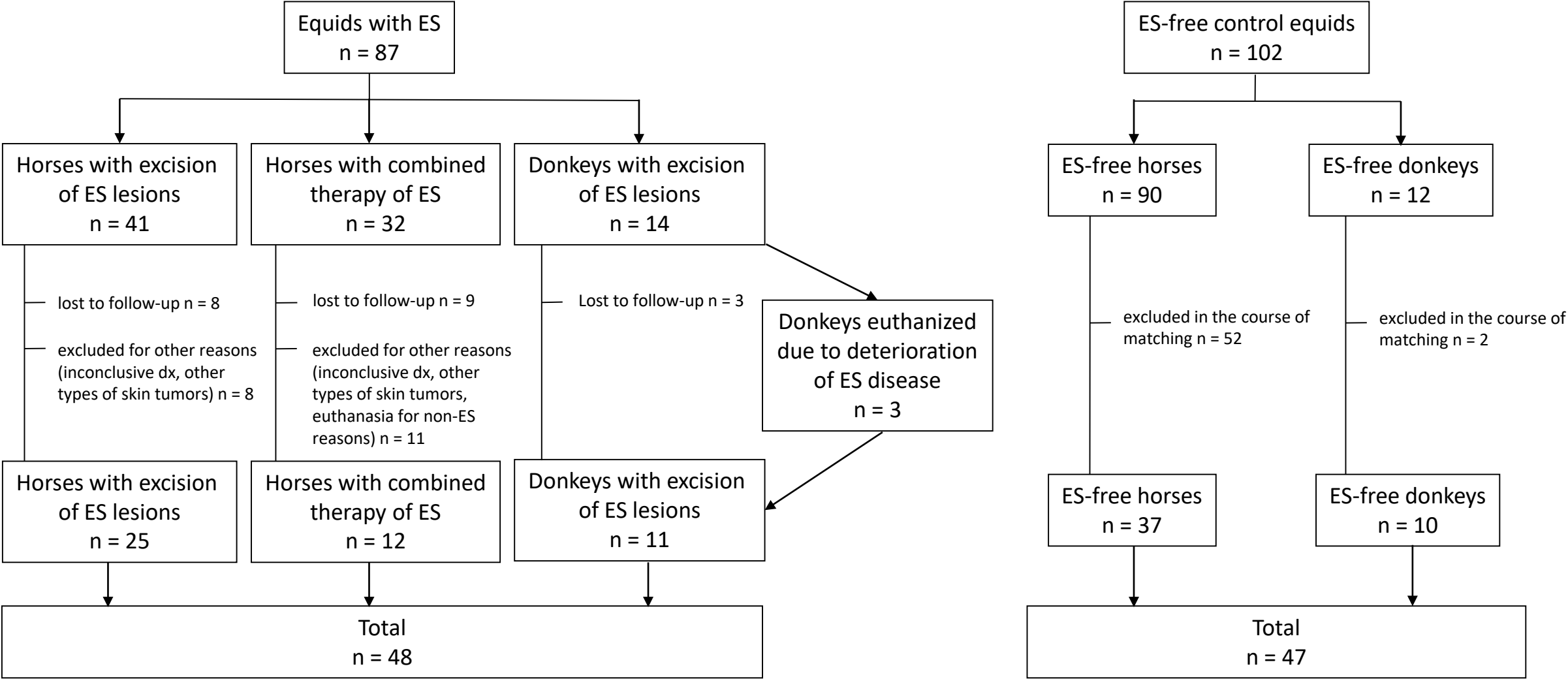

Supplement: Supplementary file 5 — Supplementary file5 (PDF 80.4 KB) [file 11259_2022_9930_MOESM5_ESM.pdf]
